# Supplementary material for: Predictive autoantibodies before the diagnosis of type I diabetes in adults
Source: PLoS One. 2026 Oct 15;21(10):e0352531. doi: 10.1371/journal.pone.0352531 (PMC13671519; doi:10.1371/journal.pone.0352531)
Supplement: Supplementary file 1 [file pone.0352531.s001.docx]

Incident Adult Onset Autoimmune Diabetes Diagnoses By Year

|  |  |
| --- | --- |
| 2009 | 124 |
| 2010 | 108 |
| 2011 | 123 |
| 2012 | 122 |
| 2013 | 80 |
| 2014 | 73 |
| 2015 | 108 |
| 2016 | 151 |
| 2017 | 120 |
| 2018 | 141 |

**S1 Fig. Annual incidence of adult-onset type 1 diabetes (AOT1D) in the U.S. Military Health System, 2009–2018.** Bar graph showing the estimated number of new AOT1D cases per year, used to calculate the average annual incidence of AOT1D.

**S2 Fig. Autoantibodies against ZNT8-W in the AOT1D cohort.** Autoantibody levels were determined by LIPS against ZNT8-W. Each symbol represents a sample from a healthy control subject (CTRL) or with a final diagnosis of AOT1D. Autoantibody levels are plotted in light units (LU) on a log^10^ scale, and the dashed lines represent the cut-off level for determining seropositive autoantibodies for each autoantigen based on the mean plus 3 standard deviations of the controls.
