# Supplementary material for: Predictive autoantibodies before the diagnosis of type I diabetes in adults
Source: PLoS One. 2026 Oct 15;21(10):e0352531. doi: 10.1371/journal.pone.0352531 (PMC13671519; doi:10.1371/journal.pone.0352531)
Supplement: Supplementary file 2 [file pone.0352531.s002.docx]

**S2 Fig. Autoantibodies against ZNT8-W in the AOT1D cohort.** Autoantibody levels were determined by LIPS against ZNT8-W. Each symbol represents a sample from a healthy control subject (CTRL) or with a final diagnosis of AOT1D. Autoantibody levels are plotted in light units (LU) on a log^10^ scale, and the dashed lines represent the cut-off level for determining seropositive autoantibodies for each autoantigen based on the mean plus 3 standard deviations of the controls.
